# Circulating brain-enriched microRNAs as novel biomarkers for detection and differentiation of neurodegenerative diseases

**DOI:** 10.1186/s13195-017-0316-0

**Published:** 2017-11-09

**Authors:** Kira S. Sheinerman, Jon B. Toledo, Vladimir G. Tsivinsky, David Irwin, Murray Grossman, Daniel Weintraub, Howard I. Hurtig, Alice Chen-Plotkin, David A. Wolk, Leo F. McCluskey, Lauren B. Elman, John Q. Trojanowski, Samuil R. Umansky

**Affiliations:** 1 DiamiR LLC, Princeton, NJ 08540 USA; 2 Department of Neurology, Perelman School of Medicine, University of Pennsylvania, Philadelphia, PA 19104 USA; 3 Department of Psychiatry, Perelman School of Medicine, University of Pennsylvania, Philadelphia, PA 19104 USA; 4 Institute on Aging, Center for Neurodegenerative Disease Research, Department of Pathology and Laboratory Medicine, Perelman School of Medicine, University of Pennsylvania, Philadelphia, PA 19104 USA; 5 Present address: Department of Neurology, Houston Methodist Hospital, Houston, TX 77030 USA

**Keywords:** Alzheimer’s disease, Frontotemporal dementia, Parkinson’s disease, Amyotrophic lateral sclerosis, microRNA, Blood-based biomarkers

## Abstract

**Background:**

Minimally invasive specific biomarkers of neurodegenerative diseases (NDs) would facilitate patient selection and disease progression monitoring. We describe the assessment of circulating brain-enriched microRNAs as potential biomarkers for Alzheimer’s disease (AD), frontotemporal dementia (FTD), Parkinson’s disease (PD), and amyotrophic lateral sclerosis (ALS).

**Methods:**

In this case-control study, the plasma samples were collected from 250 research participants with a clinical diagnosis of AD, FTD, PD, and ALS, as well as from age- and sex-matched control subjects (*n* = 50 for each group), recruited from 2003 to 2015 at the University of Pennsylvania Health System, including the Alzheimer’s Disease Center, the Parkinson’s Disease and Movement Disorders Center, the Frontotemporal Degeneration Center, and the Amyotrophic Lateral Sclerosis Clinic. Each group was randomly divided into training and confirmation sets of equal size. To evaluate the potential of circulating microRNAs enriched in specific brain regions affected by NDs and present in synapses as biomarkers of NDs, the levels of 37 brain-enriched and inflammation-associated microRNAs in the plasma of all participants were measured using individual qRT-PCR. A “microRNA pair” approach was used for data normalization.

**Results:**

MicroRNA pairs and their combinations (classifiers) capable of differentiating NDs from control and from each other were defined using independently and jointly analyzed training and confirmation datasets. AD, PD, FTD, and ALS are differentiated from control with accuracy of 0.89, 0.90, 0.88, and 0.83 (AUCs, 0.96, 0.96, 0.94, and 0.93), respectively; NDs are differentiated from each other with accuracy ranging from 0.77 (AUC, 0.87) for AD vs. FTD to 0.93 (AUC, 0.98) for AD vs. ALS. The data further indicate sex dependence of some microRNA markers. The average increase in accuracy in distinguishing ND from control for all and male/female groups is 0.06; the largest increase is for ALS, from 0.83 for all participants to 0.92/0.98 for male/female participants.

**Conclusions:**

The work presented here suggests the possibility of developing microRNA-based diagnostics for detection and differentiation of NDs. Larger multicenter clinical studies are needed to further evaluate circulating brain-enriched microRNAs as biomarkers for NDs and to investigate their association with other ND biomarkers in clinical trial settings.

**Electronic supplementary material:**

The online version of this article (doi:10.1186/s13195-017-0316-0) contains supplementary material, which is available to authorized users.

## Background

Neurodegenerative diseases (NDs) have become a serious medical and social problem in developed countries, owing to significantly increased lifespans. For example, Alzheimer’s disease (AD) is now the fifth leading cause of death for those aged 65 years and older in the United States [1]. Despite intense research and numerous clinical trials, no effective treatments are currently available for NDs. One key element in the development of new therapies is the availability of diagnostic tools to help identify disease early, stratify patients for clinical trials, and monitor disease progression during treatment. In this respect, the most advanced diagnostic approaches for NDs, such as analysis of proteins in cerebrospinal fluid (CSF) and various imaging techniques [2, 3], are highly promising but not suitable for primary screening and monitoring purposes, owing to their invasiveness and high cost. Minimally invasive, cost-effective biomarkers of NDs would be very helpful in the advancement of ND diagnosis and treatment [4–8]. Several factors complicate the search for such biomarkers [7, 9]. First, mechanisms of ND initiation and development are not well understood; second, NDs may develop without any signs or symptoms for 10–20 years prior to clinical manifestation; and third, commonly occurring comorbidities and symptomatic overlap, such as cognitive impairment, complicate the differential diagnosis of NDs (*see* [10, 11] for detailed discussion of the need and the roadmap for the development of blood-based biomarkers for AD and other dementias).

Recently, several groups have proposed the use of microRNAs (miRNAs) circulating in plasma or serum for ND detection [12–23]. miRNAs are small molecules (~22 nucleotides) that play important roles in gene regulation by binding to complementary regions of messenger transcripts and repressing their translation or regulating their degradation [24, 25]. On the basis of sequence complementarity analysis, an individual miRNA can bind to and regulate > 100 messenger RNAs (mRNAs), and an mRNA can be regulated by multiple miRNAs; thus, as potential biomarkers, miRNAs are reflective of multiple cellular processes. Over 2000 miRNAs have been discovered in human cells to date, and many of these miRNAs are specific to or overexpressed in certain organs, tissues, and cells [25–30]. Some miRNAs, including those that are cell-specific, can be enriched in particular cellular compartments, such as neurites and synapses [31–37]. miRNAs can be secreted or excreted into the extracellular space [38–41] and are detectable in plasma and serum [14, 42–44].

Intracellular concentrations and rates of secretion of miRNAs can be dramatically affected by physiological and pathological cellular processes [39, 45–47]. In the case of mild cognitive impairment (MCI), a heterogeneous syndrome characteristic of early stages of various NDs, we have previously shown that the condition can be detected by analysis of miRNAs enriched in synapses of brain regions affected in early AD, such as the hippocampus [14, 48]. The concentration of a brain-enriched miRNA in bodily fluids depends on intrinsic factors such as its expression; its intracellular localization; and disease-associated changes in expression, metabolism, and secretion. It also depends on extrinsic factors such as blood supply to a particular brain area, changes in blood-brain barrier permeability, and miRNA stability in circulation.

To compensate for the impact of factors unrelated to a specific ND and to account for smaller changes in miRNA concentrations accompanying slowly developing pathologies (as compared with those in acute diseases such as stroke), other brain-enriched miRNAs can be used for normalization of the miRNA biomarkers. This miRNA “pair” approach (*see below*) has led to the discovery of miR-132 and miR-134 families of miRNA biomarker pairs capable of differentiating MCI from age-matched controls with approximately 0.90 accuracy [48, 49]. Interestingly, these miRNA pairs proved less effective as biomarkers for detecting AD dementia stages [49], possibly owing to the loss of synapses and death of neurons in the hippocampus causing the amount of hippocampal synaptic miRNAs to decrease in plasma as the disease progresses.

The present study was designed to evaluate circulating brain-enriched miRNAs as potential biomarkers for detection and differentiation of AD, Parkinson’s disease (PD), frontotemporal lobar degeneration (frontotemporal dementia [FTD]), and amyotrophic lateral sclerosis (ALS).

## Methods

### Clinical diagnosis and plasma samples

Thirty-seven miRNAs were analyzed in two 0.5-ml aliquots (1 ml total) of K_2_-ethylenediaminetetraacetic acid-treated frozen plasma samples from 250 research participants (*n* = 50 for each ND) recruited and clinically evaluated at the University of Pennsylvania Health System, including the Alzheimer’s Disease Center (in 2003–2014), the Parkinson’s Disease and Movement Disorders Center (in 2010–2015), the Frontotemporal Degeneration Center (in 2010–2013), and the Amyotrophic Lateral Sclerosis Clinic (in 2010–2014). Fifty samples from age- and sex-matched control subjects were collected in 2009–2015. The ND conditions were clinically diagnosed according to published procedures [50–58]; demographic characteristics of the study groups, including control subjects, are summarized in Table 1 and Additional file 1. The study participants in the AD group were selected on the basis of AD phenotype and tau and amyloid biomarkers as determined by CSF analysis. The biofluid samples were collected and processed on the day they were obtained following the same standard operating procedures in all specialty clinical practices at the Center for Neurodegenerative Disease Research, University of Pennsylvania [59]. Blood samples were centrifuged at 3000 × *g* for 15 minutes at 4 °C and aliquoted into 2.0-ml polypropylene cryovials (Corning Life Sciences, Corning, NY, USA). The aliquots were stored at −80 °C. De-identified samples were sent to the Asuragen laboratory (Austin, TX, USA) for RNA isolation and qRT-PCR (*see below*). Clinical information, including demographics, diagnosis and cognitive scores, and identifiers to match the samples, were sent to the DiamiR principal investigator, who kept the information blinded from the laboratory personnel.

**Table 1:** Cohort demographics and clinical data

Diagnosis	Subgroups	No. of subjects	Age (years)	Sex (M/F)	MMSE [NR]	CSF Aβ_42_	CSF t-tau	CSF p-tau	Years from diagnosis	Years of education
Control	N/A	50	64.06 ± 9.8 (25–83)	24/26	29.3 ± 1.0	N/A	N/A	N/A	N/A	16.44 ± 2.7
AD	N/A	50	67.8 ± 10.4 (53–87)	24/26	21.0 ± 5.6	145.7 ± 34.8	116.2 ± 68	45 ± 23.7	3.6 ± 2.2	16.5 ± 2.3
PD	Total	50	66.76 ± 7.7 (49–82)	35/15	28.9 ± 1.1 [11]	261.8 ± 49.8	43 ± 13.7	20.9 ± 7.4	10 ± 5.1	16.54 ± 2.2
	Dementia	15	72.33 ± 6.1 (61–82)	11/4	27 [1]	N/A	N/A	N/A	11 ± 3.9	16.33 ± 2.8
	MCI	8	68.37 ± 7.0 (54–77)	5/3	29 ± 0.8 [4]	N/A	N/A	N/A	10.4 ± 7.4	16.25 ± 1.7
FTD	Total	50	63.14 ± 6.9 (46–76)	28/22	24.1 ± 5.3 [46]	271.4 ± 52.3	62.2 ± 27.9	16.5 ± 7.8	3.2 ± 2.3	15.96 ± 3.1
	bv	23	61.69 ± 6.3 (46–74)	15/8	25.3 ± 4.4	278.6 ± 52.3	57.2 ± 22.5	14.5 ± 4.6	2.7 ± 2.2	16.43 ± 3.0
	PPA/Lgpen	1	70	1/0	22				2	18
	PPA/PNFA	8	67.75 ± 4.6 (61–75)	3/5	19.6 ± 9.18 [5]	269.9 ± 63.5	75.2 ± 35.2	14.6 ± 4.3	4.5 ± 2.1	15.2 ± 3.84
	PPA/SD	8	61.25 ± 8.4 (49–76)	4/4	22.1 ± 5.19	286 ± 48.4	79.9 ± 36.6	15.9 ± 4.5	4 ± 3.0	17 ± 2.82
	PSP	10	63.6 ± 7.51 (54–75)	5/5	25.6 ± 4.4	244.7 ± 46.0	50.6 ± 19.5	14.5 ± 4.6	2.7 ± 1.6	14.4 ± 2.99
ALS	Total	50	59.64 ± 10.8 (29–83)	36/14	24.8 ± 7.8 [16]	240.4 ± 76.3	71.1 ± 50.7	13.8 ± 6.8	1.8 ± 1.8	14.71 ± 2.9
	With FTD	5	65.4 ± 9.18 (49–70)	4/1	14. 3 ± 12.0 [3]	222.4 ± 88.8	107 ± 62.1	19 ± 10.1	4 ± 4.4	15.8 ± 2.3
	With MCI	3	57 ± 5.2 (54–63)	2/1	30 [1]	340 ± 3	66 ± 32	11.3 ± 0.6	1 ± 1	13.5 ± 7.8

*Abbreviations: Aβ*_*42*_ Amyloid-β 42, *AD* Alzheimer’s disease, *ALS* Amyotrophic lateral sclerosis, *FTD* Frontotemporal dementia, *PD* Parkinson’s disease, *M/F* Male/female, *MMSE* Mini Mental State Examination, *NR* Number of research participants with Mini Mental State Examination reported (MMSE is reported for all participants in a subgroup [e.g., control] if no NR value is given), *N/A* Not available, *bv* Behavioral variant, *PPA/Lgpen* Primary progressive aphasia, logopenic variant, *PPA/PNFA* Primary progressive aphasia/progressive nonfluent aphasia, *PPA/SD* Primary progressive aphasia/semantic dementia, *PSP* Progressive supranuclear palsy, *CSF* Cerebrospinal fluid, *MCI* Mild cognitive impairment, *p-tau* Phosphorylated tau, *t-tau* Total tau

All groups were randomly divided into two sets of equal size (25 samples each, balanced by age, sex, and comorbidities) and used in training and confirmation experiments. The PD and ALS groups had fewer female participants, and the ALS group was younger than other ND and control groups. Age may affect miRNA expression, and thus differences in the age composition of different groups may contribute to the differentiation of the groups with specific miRNAs. In the present study, however, the average ages were not significantly different between various groups (*see* Additional file 1); we found that the exclusion of several outliers did not change the results. Correlations between miRNA ratios and age in all pathology cohorts are low (*r* < 0.2) and have low statistical significance (*p* > 0.04). To determine the source of miRNA ratio differences in disease groups and the control group, we performed linear regression analysis of miRNA ratios using disease group identity and age as covariates. *p* Values for the significance of the disease group identity for all miRNA pairs selected were < 0.0001, indicating that the differentiation of two groups depended on diagnosis. Only in the ALS group did some miRNA pairs correlate with age with *p* < 0.05. Age adjustment for these pairs slightly increased the accuracy (1–2%); age adjustment for all other pairs either did not change or slightly improved the accuracy (by < 2%). Similarly, to determine the effect of sex on differentiation of PD and ALS (the two NDs with smaller numbers of female participants; *see* Table 1 and Additional file 1) from control subjects, we performed a linear regression analysis of miRNA ratios using sex as a covariate. For most pairs, the effect of sex adjustment was insignificant. Additional stratified analysis of all NDs (described in the “miRNA biomarker sex-dependent effects” subsection of the Results section below) revealed miRNA pairs differentiating male and female participants separately better than all (male and female) participants.

### RNA isolation and qRT-PCR

RNA was extracted from 1 ml of plasma using a TRIzol treatment (Life Technologies, Carlsbad, CA, USA) and silica (Ambion Glass Fiber Microcolumn; Fisher Scientific, Pittsburgh, PA, USA) binding protocol (http://asuragen.com/wp-content/uploads/2016/05/biomarkers.pdf). Single-target qRT-PCR was performed using the TaqMan® Reverse Transcription Kit and miRNA-specific stem-loop primers (Applied Biosystems, Foster City, CA, USA). The RT step for miRNA measurements was performed in triplicate and each RT reaction was then used separately for PCR. 2µl plasma equivalents were present in the final PCR. (Additional file 2 presents the qRT-PCR variability for each tested miRNA.) Placental RNA was used as a positive control and no template as a negative control. Calibration curves for each miRNA were generated to facilitate comparing and combining of training and confirmation datasets. Quality control of miRNA preparations was performed by testing two ubiquitous miRNAs, miR-16 and miR-27a, in each plasma preparation; all samples with values within 2 SD of the average value were qualified as acceptable for analysis. The average SDs of miR-16 and miR-27a were 1.40 and 1.01 in the training and confirmation sets, respectively. miRNAs with cycle threshold (*C*_t_) > 37 were excluded from the analysis of a respective sample. Although small RNA yield was not directly measured after RNA extraction, the control data reported in Additional file 2 provide a good indicator of miRNA yield.

### Statistical analyses

In our approach, an effective biomarker is a ratio (pair) of two brain-enriched miRNAs. Software developed at DiamiR [48, 49] was used for all calculations, including determination of miRNA pairs capable of differentiating NDs from controls and from each other on the basis of their concentration ratios (∆*C*_t_) in plasma. The application was designed using .NET technology with a set of .NET statistical packages. The selection of miRNA pairs as well as their combinations was based on the AUC and *p* values as follows. For every pair, ROC curves were constructed to calculate the area under the ROC curve (AUC). In the training set analysis, pairs with AUC ≥ 0.75 for AD, PD, and ALS, as well as AUC ≥ 0.70 for FTD, were initially accepted. Training and confirmation sets were of the same size. A lower cutoff for FTD was used because of high heterogeneity of the group (presence of several FTD syndromes). In the combined set, there were 23 behavioral variant frontotemporal dementia (bvFTD), 17 primary progressive aphasia (PPA), and 10 progressive supranuclear palsy (PSP) cases. The acceptance *p* value threshold was set on the basis of significance of differences between any two experimental groups, calculated by the Mann-Whitney *U* test with Bonferroni correction. Validation of miRNA pair selection and testing for overfitting were performed using random forest classification with bootstrapping. For 37 miRNAs used, the total number of pairs was 666 (36 × 37/2). The ratios A/B and B/A are equivalent as potential biomarkers because they have the same variance, difference of means, correlation coefficients, *p* values, and ROC parameters. In cases where disease was compared with control, the ratio whose numerical value was larger in disease samples was selected. According to the Bonferroni correction, statistically significant results should then have a *p* value of 0.05/666 = 7.5 × 10^−5^. Because we were using highly correlated miRNAs for creating pairs (*C*_t_ Spearman’s rank correlation coefficient, ≥ 0.8), the number of pairs analyzed was reduced to about 160, and thus the threshold for statistical significance rose to ~ 3.0 × 10^−4^. Pair sensitivity and specificity are reported for the cutoff points on the ROC curves that provided the best overall accuracy. For selecting effective pair combinations, miRNA classifiers, a stepwise algorithm for logistic regression using a linear model with no interaction [60] was applied. The miRNA classifiers having AUC ≥ 0.9 for AD, PD, and ALS, as well as AUC ≥ 0.8 for FTD, in the combined (training + confirmation) set were accepted.

## Results

### miRNA selection

In this work, we performed targeted preselection of miRNAs present in synapses, enriched in different brain regions, and also detectable in plasma. The levels of all preselected miRNAs in plasma samples were determined by qRT-PCR. The miRNAs analyzed in the study are listed in Additional file 3 and included (1) miRNAs present in synapses [33, 35, 36, 61, 62] and enriched in different brain regions affected by the target pathologies [26–30, 63–67]; (2) miRNAs associated with inflammatory processes [68–71]; (3) miR-206 highly enriched in muscle tissue and in cerebellum [68, 72, 73]; (4) ubiquitous apoptosis-associated miR-16 [74]; and (5) miR-451, which is more effectively excreted from pathologic than normal cells [39]. As shown in Additional file 3, certain brain-enriched miRNAs are expressed in several brain regions; typically, the levels of these miRNAs in specific brain regions (shown in bold in Additional file 3) are significantly higher than in others. Potential roles of many of the miRNAs listed in Additional file 3 in NDs are being investigated by several groups [62, 75–88].

### ND detection

**Fig. 1:**
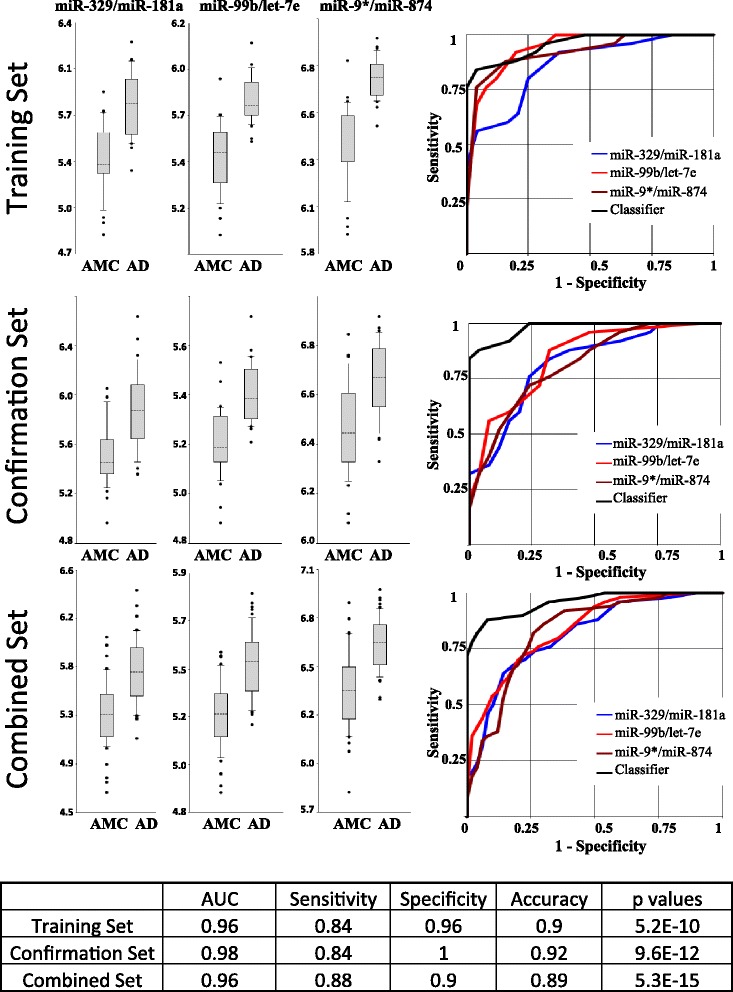
Differentiation of Alzheimer’s disease (AD) from control by select microRNA (miRNA, miR) pairs and their combination. The performance of three miRNA pairs and resulting signature in the training, confirmation, and combined sets is shown. The table at the bottom of the figure indicates performance of the miRNA classifier in the three sets. The area under the ROC curve (AUC) is presented; sensitivity, specificity, and accuracy of each biomarker/normalizer pair were calculated at the cutoff point with the highest accuracy. In the box-and-whisker plots, the ratios are calculated as 2^−ΔCt^ × 100, and the results are presented in log_10_ scale. The upper and lower limits of the boxes and the lines inside the boxes indicate the 75th and 25th percentiles and the median, respectively. The upper and lower horizontal bars denote the 90th and 10th percentiles, respectively. The points indicate assay values located outside 80% of data. *AMC* Age- and sex-matched controls

**Fig. 2:**
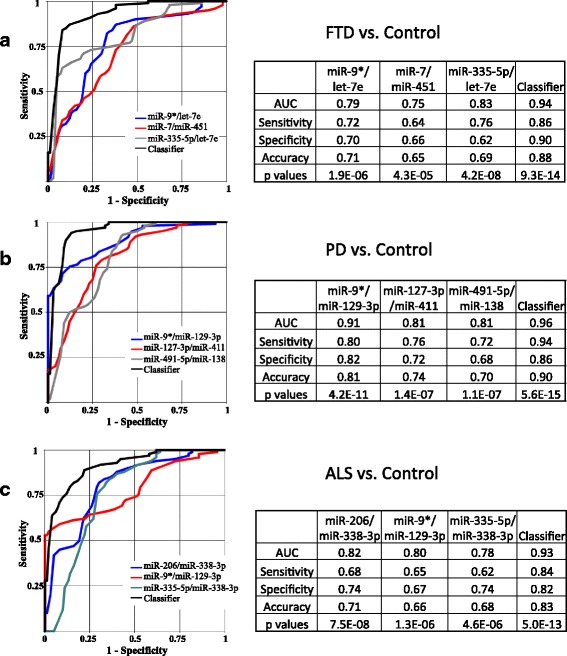
Differentiation of neurodegenerative diseases (NDs) from control in combined set by select microRNA (miR) pairs and their combinations. **a** Frontotemporal dementia (FTD) vs. control. **b** Parkinson’s disease (PD) vs. control. **c** Amyotrophic lateral sclerosis (ALS) vs. control. AUC is area under ROC curve

As described above, each group (AD, FTD, PD, ALS, and age- and sex-matched control group) was divided in two equal sets and analyzed independently in the training and confirmation studies. miRNA pairs and their combinations (miRNA classifiers) capable of differentiating each ND from controls with the highest accuracy were assessed in the training set and verified in the confirmation set analysis, followed by the analysis of the combined dataset (Figs. 1 and 2 and Additional file 4). Combining two or more qRT-PCR experiments is often challenging because of possible technical variations in RNA extraction, RT and/or PCR kits, and instrumental fluctuations. Furthermore, samples for the confirmation study were kept frozen for an additional 6 months. Nonetheless, certain miRNA pairs and classifiers effectively differentiated AD, FTD, PD, and ALS from controls in both training and confirmation studies (Additional file 4). The accuracy of ND detection in the combined dataset by miRNA classifiers for AD, PD, FTD, and ALS was 0.89, 0.90, 0.88, and 0.83, respectively. The comparison of the differentiation obtained with individual miRNAs and with miRNA pairs is reported in Additional file 5. Only muscle-enriched miR-206, whose average concentration is about eight times higher in patients with ALS than in control subjects, differentiates these two groups with accuracy similar to that of miRNA pairs. In all other cases, the use of miRNA pairs produces much better differentiation between NDs and controls than individual miRNAs.

### ND differentiation

**Fig. 3:**
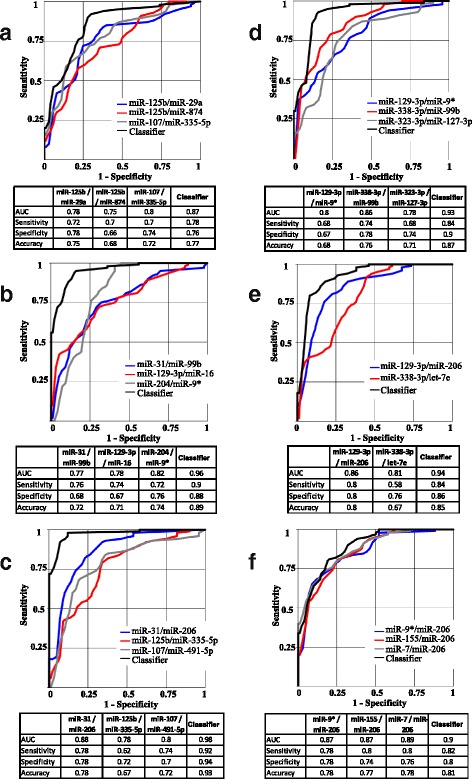
Differentiation of neurodegenerative diseases (NDs) in combined set by select microRNA (miR) pairs and their combinations. **a** Alzheimer’s disease (AD) vs. frontotemporal dementia (FTD). **b** Alzheimer’s disease (AD) vs. Parkinson’s disease (PD). **c** AD vs. amyotrophic lateral sclerosis (ALS). **d** FTD vs. PD. **e** FTD vs. ALS. **f** PD vs. ALS

The same approach was used for establishing miRNA biomarkers capable of differentiating the four NDs (AD, PD, FTD, and ALS) from each other. Figure 3 and Additional file 6 present miRNA classifiers effectively distinguishing two NDs in the training and confirmation studies as well as in the combined dataset. The overall accuracy in the range of 0.75–0.93 was obtained. The lowest accuracy (0.77 with most effective miRNA biomarkers) was observed for differentiation of AD and FTD, for which partial anatomic overlap between the affected brain regions could be larger than between other NDs. The miRNA pairs most effectively differentiating disease groups from each other and from control are listed in Additional file 7.

#### ND subgroup differentiation

Table 1 summarizes comorbidities and subgroups of NDs included in the study. Although the sample size of individual subgroups was insufficient for obtaining statistically significant results, promising miRNA biomarker candidates capable of distinguishing FTD syndromes were identified. Data presented in Additional file 8A for differentiation between bvFTD, PPA, and PSP with accuracy in the range of 0.70–0.87 warrant further studies of larger patient cohorts. Additional file 8B and C present data on differentiation of PD with dementia from other patients with PD and on differentiation of ALS-FTD from other patients with ALS, respectively.

### miRNA biomarker sex-dependent effects

In our other studies focused on distinguishing very mild/mild cognitive impairment from control, we observed sex-specific differences in plasma concentrations of some brain-enriched miRNAs in patients and controls (unpublished data). Thus, in this study, we performed the additional stratified analysis for the four pathologies (AD, FTD, PD, and ALS) and control in the combined dataset as follows:

1. All (male and female) participants in each disease group vs. all control participants
2. Male participants in each disease group vs. male control participants
3. Female participants vs. female control participants

Table 2, section A, shows the performance of effective miRNA classifiers for differentiating each ND from control in all (male and female), male, and female groups. There are no significant differences in AUC and accuracy between these groups, which indicates that these miRNAs behave similarly in controls and male and female patients. However, other tested miRNAs demonstrated sex-associated effects, and miRNA pairs that included these miRNAs more effectively differentiated male or female participants from sex-matched controls. Table 2, section B, presents the most effective miRNA classifiers with additional miRNA biomarker candidates for differentiating AD, FTD, PD, and ALS vs. control separately in males and females. In this case, both AUC and accuracy were significantly higher than effective miRNA classifiers for all (male plus female) participants. In addition, we compared male and female subgroups in each disease group. The data presented in Additional file 9 demonstrate that certain miRNA pairs are capable of differentiating sex-specific subgroups from each other in every disease group with 66–75% accuracy. These data demonstrate sex-specific patterns in levels of circulating brain-enriched miRNAs in both patient and control samples and suggest that sex-specific assays can potentially yield higher accuracy in diagnosing NDs.

**Table 2:** Sex-dependent effect in differentiation of neurodegenerative disease s from control by microRNA classifiers

		AD-CNTR	FTD-CNTR	PD-CNTR	ALS-CNTR
A. miRNA classifiers most effectively differentiating NDs from control in all (male + female) participants					
		miR-99b/miR-181a and miR-9*/miR-874 and miR-7/miR-16	miR-335/let-7e and miR-99b/let-7e and miR-9*/miR-181a	miR-9*/miR-129-3p and miR-99b/miR-874 and miR-9*/miR-411	miR-206/miR-31 and miR-206/miR-125b and miR-99/miR-338-3p
Male + female	AUC	0.97	0.94	0.96	0.95
	Accuracy	0.91	0.91	0.86	0.83
Male	AUC	0.98	0.98	0.96	0.94
	Accuracy	0.92	0.94	0.88	0.9
Female	AUC	0.97	0.91	0.96	0.95
	Accuracy	0.9	0.88	0.85	0.83
B. miRNA classifiers most effectively differentiating NDs from control in male and female groups					
Male		miR-99b/miR-181a and miR-125/miR-874 and miR-9*/miR-29a	miR-335/let-7e and miR-99b/let-7e and miR-9*/miR-181a	miR-9*/miR-129-3p and miR-99b/miR-146a and miR-9*/miR-204	miR-206/miR-155 and miR-9*/miR-129-3p and miR-335/miR-338-3p
	AUC	0.99	0.98	0.98	0.99
	Accuracy	0.94	0.94	0.92	0.92
Female		miR-99b/miR-181a and miR-9*/miR-874 and miR-7/miR-451	miR-491/let-7e and miR-107/miR-9 and miR-28/miR-181a	miR-9*/miR-29a and miR-99b/miR-874 and miR-491/let-7e	miR-206/miR-7 ph2008and miR-9*/miR-125b and miR-491/miR-204
	AUC	0.99	0.98	0.99	0.99
	Accuracy	0.96	0.92	0.95	0.98

*Abbreviations: AD* Alzheimer’s disease, *FTD* Frontotemporal dementia, *miR* MicroRNA, *ND* Neurodegenerative disease, *PD* Parkinson’s disease, *CNTR* Control

## Discussion

Previously, we proposed a novel approach to the development of biomarkers in the area of neurodegenerative, neurodevelopmental, and neurological diseases based on analysis of brain-enriched miRNAs circulating in plasma, and we demonstrated the viability of the concept for early AD detection with the discovery of two miRNA families capable of detecting MCI and pre-MCI with high accuracy [48, 49]. In the present study, we used this approach for the detection and differentiation of four NDs: AD, PD, FTD, and ALS.

The most common approaches of searching for circulating miRNA biomarkers to detect a specific pathology are based on the analysis of as many plasma/serum miRNAs as technically feasible using miRNA arrays or next-generation sequencing, followed by qRT-PCR of the identified candidates, and normalization of miRNA(s) whose concentration significantly changes in a pathology sample per minimally variable miRNA or average of all miRNAs tested. There are several disadvantages to these approaches: (1) their sensitivity is significantly lower than that of qRT-PCR, and thus many brain-enriched miRNAs are not reliably detected in plasma, which precludes their further analysis; (2) on one hand, the variability of these methods is high, and many potential candidate miRNA biomarkers are not confirmed by qRT-PCR, and on the other hand, some promising biomarkers are not selected as candidates; and (3) many potential normalizer miRNAs are not uniformly expressed in various pathologies. The latter consideration is especially important in NDs in elderly people because there is a high chance of comorbidities as well as use of various medications. For example, plasma concentration of miR-16, which is widely used as a normalizer in other indications, is changed in patients with AD ([89, 90] and our unpublished data). Other considerations are potential changes of brain blood supply or blood-brain barrier permeability, which would affect concentrations of brain-enriched miRNAs in plasma. Thus, in our earlier studies, we developed a different approach based on miRNA pairs, consisting of one miRNA enriched in synapses of a brain region affected by the disease and another miRNA enriched in a different brain region or cell type, such as glial cells. In effective miRNA pairs, miRNAs are frequently highly correlated [49], decreasing intersubject variability. By combining two or three effective miRNA pairs into a single miRNA classifier, we achieve greater accuracy. Other groups have also identified miRNA pairs as effective biomarkers in the context of cancer diagnosis and prognosis [91–93].

The following factors, which may complicate the search for miRNA biomarkers, should be considered: (1) data on miRNA enrichment in the brain and the different brain regions is still limited, and some brain-enriched miRNAs can be expressed, although at different levels, in several brain regions; (2) ND progression can substantially change both underlying processes, such as synapse dysfunction and destruction in early disease stages and neuronal death in late disease stages, and brain regions involved in a disease due to the expansion of pathologic processes to new brain regions; (3) NDs exhibit heterogeneous clinical symptoms and different brain pathologies (in this respect, analysis of larger cohorts of pathologically homogeneous ND groups would be valuable); and (4) development of neurodegeneration can result in changes in other tissues and organ systems, such as the muscle fibers in ALS.

The data obtained in the present study support our minimally invasive approach to the detection of NDs based on the analysis of circulating brain-enriched miRNA in plasma. Individual miRNA pairs tested in this study classified diseases with accuracies > 0.80, and combinations of several miRNA pairs demonstrated accuracies of up to 0.90. Some miRNA pairs proved to be effective in detecting more than one ND, such as AD and FTD, which partially affect overlapping brain regions. Effective classifiers for ALS detection in addition to brain-enriched miRNAs included muscle-enriched miR-206, which is also highly expressed in cerebellum [72, 94].

As expected, miRNA classifiers for AD dementia were different from the optimal miRNA classifiers for MCI and pre-MCI [48, 49]. MCI is a heterogeneous syndrome characteristic of many NDs. During the progression from MCI to the dementia stage of AD, the ratio of miRNA levels in bodily fluids can change as a result of several factors. Hippocampal synapse and neurite degeneration during early stages of the disease leads to reduced numbers of synapses and neurites in later stages of AD, resulting in diminished amounts of miRNAs being excreted from synapses and neurites of the hippocampus; in addition, because of the increased neuronal cell death that occurs during the later stages of AD, the concentration of neuronal cell body miRNAs in bodily fluids increases. As the disease progresses, new brain regions become involved in the pathology, compromising the reliability of the normalizer miRNAs. This results in numerator miRNAs of effective biomarker pairs for early AD (e.g., miR-128a and miR-874) becoming denominators in effective pairs for later disease stages. Concurrently, because the pathology expands to new brain regions (e.g., cortex and midbrain), synaptic miRNAs from those regions, such as miR-107 and miR-9*, become good biomarker numerators (Additional file 4A). These events can be of informative value for disease monitoring. For example, changes in relative concentrations of miRNAs enriched in different brain regions or different cell types (e.g., neurons and glial cells) may be used as an indicator of disease progression.

Our analysis further revealed miRNA classifiers differentiating AD from FTD with accuracy > 0.75 and all other NDs from each other with accuracy in the range of 0.80–0.90, a highly promising outcome for such heterogeneous diseases, which are in many cases accompanied by other related comorbidities.

Recent data indicate that brain aging and AD development are sex-dependent phenomena [95–98]. Analysis of the data presented here reveals sex dependence of certain brain-enriched miRNAs, which distinguished NDs from control in sex-specific subsets with significantly better accuracy than in the total (female plus male) sets (Table 2, section B). The effect is particularly notable for female participants, such as that accuracy is 0.98 for female and 0.83 for all patients with ALS. More detailed, larger studies are needed to further substantiate this observation and to investigate potential physiological mechanisms underlying this phenomenon. In particular, it has to be determined whether these sex-dependent differences are due to differential expression, secretion, or excretion of some brain-enriched miRNAs, intrinsic sex-dependent differences associated with NDs, or both.

It is worth noting that the approach of the present study was based on analysis of brain-enriched miRNAs in the total pool of miRNAs extracted from plasma. Recently, exosomes, and in particular neuronally derived exosomes, have been studied as potential biomarkers of NDs [99–101]. The promise of exosomes as biomarkers of pathology is due largely to their protein content and membrane composition because there is a high degree of variability in miRNA distribution across exosome populations, and contradictory data exist on the fraction of miRNAs circulating in exosomes as compared with other forms of cell-free miRNAs in the bloodstream [47, 102, 103].

### Limitations

A limitation of this study is that all participants were recruited at a single clinical site. Larger, multicenter studies are needed to further evaluate the utility of the approach described herein. A further limitation of the study is that the blood samples were collected from symptomatic patients only. Longitudinal studies are needed to assess the prognostic value of the biomarker candidates in presymptomatic patients.

## Conclusions

We report promising data on differentiation of four NDs from control and from each other by brain-enriched miRNA classifiers detectable in plasma. This work builds on our earlier studies on brain-enriched miRNAs as biomarkers of MCI [48, 49]. miRNAs hold strong potential as effective and patient-friendly biomarkers, and several miRNA-based assays are currently used in oncology clinical practice as part of “rule-in/rule-out” diagnostic panels. Future work will address the utility and scope of application of circulating brain-enriched miRNAs as biomarkers of NDs.

## Additional files

Additional file 1: Age and sex distribution in AD, FTD, PD, and ALS groups and control. (PDF 171 kb)

Additional file 2: qRT-PCR controls and median data. (XLSX 17 kb)

Additional file 3: miRNAs analyzed in the study. (PDF 57 kb)

Additional file 4: miRNA pairs and classifiers differentiating NDs from control in training, confirmation, and combined sets. (PDF 225 kb)

Additional file 5: Comparison of the best accuracy and AUC obtained for each disease group vs. control using individual miRNAs and miRNA pairs. (XLSX 11 kb)

Additional file 6: miRNA pairs and classifiers differentiating NDs from each other in training, confirmation, and combined sets. (PDF 291 kb)

Additional file 7: miRNA pairs most effectively differentiating different groups. (XLSX 18 kb)

Additional file 8: Differentiation of ND subgroups from other research participants with the same ND. (PDF 89 kb)

Additional file 9: Differentiation of male (M) and female (F) participants within each disease group and control by miRNA pairs. (XLSX 11 kb)

## Abbreviations

Aβ_42_: Amyloid-β 42
AD: Alzheimer’s disease
ALS: Amyotrophic lateral sclerosis
AMC: Age- and sex-matched controls
bvFTD: Behavioral variant frontotemporal dementia
CNTR: Control
CSF: Cerebrospinal fluid
*C*_t_: Cycle threshold
FTD: Frontotemporal dementia
MCI: Mild cognitive impairment
miRNA/miR: MicroRNA
MMSE: Mini Mental State Examination
mRNA: Messenger RNA
N/A: Not available
ND: Neurodegenerative disease
PD: Parkinson’s disease
PPA: Primary progressive aphasia
PPA/Lgpen: Primary progressive aphasia, logopenic variant
PPA/PNFA: Primary progressive aphasia/progressive nonfluent aphasia
PPA/SD: Primary progressive aphasia/semantic dementia
PSP: Progressive supranuclear palsy, p-tau, Phosphorylated tau
t-tau: Total tau
