# Supplementary material for: Circulating brain-enriched microRNAs as novel biomarkers for detection and differentiation of neurodegenerative diseases
Source: Alzheimers Res Ther. 2017 Nov 9;9:89. doi: 10.1186/s13195-017-0316-0 (PMC5679501; doi:10.1186/s13195-017-0316-0)
Supplement: Supplementary file 1 — Age and sex distribution in AD, FTD, PD, and ALS groups and control. (PDF 171 kb) [file 13195_2017_316_MOESM1_ESM.pdf]

Set	Group	Female + Male					Female					Male				
		N	Age Mean	Age STD	Age Min	Age Max	N	Age Mean	Age STD	Age Min	Age Max	N	Age Mean	Age STD	Age Min	Age Max
Training set	**Control**	25	64.84	7.49	51	80	13	64.92	7.32	52	78	12	64.75	8.00	51	80
	**AD**	25	67.84	10.22	53	85	13	66.62	11.84	53	85	12	69.17	8.45	56	83
	**FTD**	25	63.04	7.24	46	76	12	62.25	7.81	46	72	13	63.77	6.92	54	76
	**PD**	25	67.08	7.63	54	82	7	71.00	3.87	65	77	18	65.56	8.25	54	82
	**ALS**	25	59.80	9.99	39	78	7	59.86	6.91	50	70	18	59.78	11.13	39	78
Confirmation set	**Control**	25	65.08	11.81	25	83	13	59.30	11.81	25	70	12	71.33	8.37	57	83
	**AD**	25	67.68	10.82	54	87	13	66.84	11.51	54	84	12	68.58	10.46	56	87
	**FTD**	25	63.24	6.67	49	75	10	64.60	7.01	49	74	15	62.33	6.51	52	75
	**PD**	25	66.44	7.96	49	81	8	66.12	6.05	57	77	17	66.58	8.89	49	81
	**ALS**	25	59.48	11.71	29	83	7	59.57	13.24	40	83	18	59.44	11.48	29	74
Combined set	**Control**	50	64.96	9.79	25	83	26	62.12	10.04	25	78	24	68.04	8.69	51	83
	**AD**	50	67.76	10.42	53	87	26	66.73	11.44	53	85	24	68.88	9.30	56	87
	**FTD**	50	63.14	6.89	46	76	22	63.32	7.38	46	74	28	63.00	6.62	52	76
	**PD**	50	66.76	7.73	49	82	15	68.40	5.58	57	77	35	66.06	8.46	49	82
	**ALS**	50	59.64	10.77	29	83	14	59.71	10.15	40	83	36	59.61	11.15	29	78

Additional file 1. Age and sex distribution in AD, FTD, PD, ALS groups and control
