# Supplementary material for: Circulating brain-enriched microRNAs as novel biomarkers for detection and differentiation of neurodegenerative diseases
Source: Alzheimers Res Ther. 2017 Nov 9;9:89. doi: 10.1186/s13195-017-0316-0 (PMC5679501; doi:10.1186/s13195-017-0316-0)
Supplement: Supplementary file 3 — miRNAs analyzed in the study. (PDF 57 kb) [file 13195_2017_316_MOESM3_ESM.pdf]

miRNA	Brain enrichment	Enriched in brain regions/other	Present in synapses
Let-7e	+	**MB, PG**, Cer, FC	+
miR-107	+	**FC**, Hip, MB, PG	+
miR-125b	+	FC, MB, PG, Hip	+
miR-127-3p	+	**PG**, MB, FC	N/D
miR-128a	+	Hip, FC, HPT	+
miR-129-3p	+	**FC, MB**, Hip, TL	L/D
miR-133b	-	N/D; muscles	N/D
miR-134	+	MB, Hip, PG	+
miR-138	+	Brain-ubiquitous; axons	+
miR-146a	-	N/D; inflammatory	N/D
miR-155	-	N/D; inflammatory	N/D
miR-16	-	PG; ubiquitous	L/D
miR-181a	+	MB, FC, Hip	L/D
miR-204	+	PG, MB, Hip	+
miR-206	-	Cer; muscles	L/D
miR-218	+	**Hip**, PG, SC	+
miR-29a	-	PG, MB, FC, Hip	L/D
miR-31	-	N/D; inflammatory	N/D
miR-323-3p	+	FC, MB	+
miR-329	+	**PG, MB**	L/D
miR-335a-5p	+	**PG**, Hip	L/D
miR-338-3p	+	MB, FC, Hip, Cer, SC	N/D
miR-370	+	GC, PG	+
miR-382	+	Hip, FC	+
miR-411	+	**PG**, FC, Hip	L/D
miR-433	+	PG, MB	N/D
miR-451	-	PG, HPT, SC; ubiquitous	N/D
miR-487b	+	**PG**, MB, FC	L/D
miR-491-5p	+	**MB**, FC	+
miR-495	+	Hip	+
miR-539	+	MB, FC	N/D
miR-7	+	**PG**, FC, Hip	+
miR-874	+	Cer, Hip	+
miR-9	+	FC, MB, Hip, Cer	L/D
miR-99b	+	MB, PG, FC, Cer, Hip	L/D
miR-181a-2*	+	N/D	+
miR-9*	+	MB, Cer, Hip, FC	+

Additional file 3. miRNAs analyzed in the study
Cer – Cerebellum; FC – Frontal Cortex; Hip – Hippocampus; HPT – Hypothalamus; MB – Midbrain; PG – Pituitary Gland, TL – Temporal lobe, SC – Spinal cord. **Bold** indicates regions of maximal enrichment. L/D – Limited or contradictory data, N/D – No data.
