# Supplementary material for: Circulating brain-enriched microRNAs as novel biomarkers for detection and differentiation of neurodegenerative diseases
Source: Alzheimers Res Ther. 2017 Nov 9;9:89. doi: 10.1186/s13195-017-0316-0 (PMC5679501; doi:10.1186/s13195-017-0316-0)
Supplement: Supplementary file 4 — miRNA pairs and classifiers differentiating NDs from control in training, confirmation, and combined sets. (PDF 225 kb) [file 13195_2017_316_MOESM4_ESM.pdf]

Set	Training				Confirmation				Combined			
	SENS	SPEC	ACCUR	AUC	SENS	SPEC	ACCUR	AUC	SENS	SPEC	ACCUR	AUC
**miR-433 / miR-181a**	0.6	0.8	0.7	0.76	0.68	0.72	0.7	0.81	0.8	0.64	0.72	0.77
**miR-329 / miR-874**	0.72	0.88	0.8	0.86	0.64	0.48	0.56	0.67	0.8	0.63	0.72	0.75
**miR-329 / miR-181a**	0.8	0.75	0.78	0.86	0.64	0.76	0.7	0.82	0.64	0.86	0.75	0.82
**miR-329 / let-7e**	0.84	0.83	0.84	0.91	0.64	0.52	0.58	0.59	0.72	0.69	0.71	0.77
**miR-329 / miR-146a**	0.76	0.71	0.73	0.78	0.72	0.6	0.66	0.76	0.64	0.78	0.71	0.76
**miR-329 / miR-411**	0.52	0.83	0.67	0.71	0.76	0.72	0.74	0.83	0.7	0.73	0.72	0.76
**miR-127-3p / miR-181a**	0.64	0.76	0.7	0.74	0.76	0.76	0.76	0.83	0.64	0.74	0.69	0.75
**miR-99b / miR-874**	0.92	0.6	0.76	0.79	0.48	0.6	0.54	0.73	0.9	0.54	0.72	0.76
**miR-99b / miR-181a**	0.6	0.8	0.7	0.76	0.8	0.84	0.82	0.94	0.68	0.84	0.76	0.86
**miR-99b / let-7e**	0.88	0.8	0.84	0.94	0.72	0.72	0.72	0.84	0.7	0.8	0.75	0.84
**miR-99b / miR-128a**	0.76	0.64	0.7	0.79	0.52	0.72	0.62	0.76	0.7	0.72	0.71	0.76
**miR-107 / miR-181a**	0.76	0.76	0.76	0.85	0.68	0.72	0.7	0.85	0.78	0.68	0.73	0.79
**miR-107 / let-7e**	1	0.84	0.92	0.97	0.36	0.52	0.44	0.52	0.92	0.56	0.74	0.79
**miR-107 / miR-146a**	0.56	0.88	0.72	0.81	0.68	0.72	0.7	0.83	0.7	0.76	0.73	0.81
**miR-9* / miR-16**	0.76	0.6	0.68	0.73	0.76	0.68	0.72	0.83	0.7	0.7	0.7	0.76
**miR-9* / miR-874**	0.8	0.92	0.86	0.92	0.72	0.72	0.72	0.81	0.84	0.72	0.78	0.83
**miR-9* / miR-29a**	0.72	0.6	0.66	0.67	0.84	0.84	0.84	0.93	0.78	0.74	0.76	0.79
**miR-9* / miR-155**	0.88	0.72	0.8	0.76	0.76	0.72	0.74	0.86	0.7	0.72	0.71	0.79
**miR-7 / miR-16**	0.92	0.8	0.86	0.93	0.68	0.72	0.7	0.87	0.8	0.74	0.77	0.81
**miR-7 / miR-874**	0.68	0.92	0.8	0.84	0.6	0.64	0.62	0.68	0.72	0.68	0.7	0.75
**miR-7 / miR-451**	0.8	0.88	0.84	0.93	0.6	0.64	0.62	0.68	0.86	0.56	0.71	0.79
**miR-125b / miR-874**	0.88	0.72	0.8	0.89	0.72	0.68	0.7	0.7	0.7	0.76	0.73	0.8
**miR-107 / miR-146a & miR-9* / miR-155**	0.76	0.84	0.8	0.9	0.88	0.84	0.86	0.92	0.84	0.8	0.82	0.9
**miR-99b / miR-874 & miR-9* / miR-155**	0.88	0.92	0.9	0.94	0.88	0.8	0.84	0.9	0.86	0.92	0.89	0.91
**miR-9* / miR-16 & miR-7 / miR-874**	0.84	0.92	0.88	0.97	0.92	0.88	0.9	0.95	0.86	0.94	0.9	0.96
**miR-329 / miR-181a & miR-9* / miR-16 & miR-7 / miR-874**	0.84	0.92	0.88	0.95	0.84	0.88	0.86	0.94	0.92	0.78	0.85	0.95
**miR-107 / miR-181a & miR-9* / miR-16 & miR-7 / miR-874**	0.84	0.88	0.86	0.95	0.92	0.8	0.86	0.94	0.92	0.78	0.85	0.95
**miR-107 / miR-146a & miR-9* / miR-16 & miR-7 / miR-874**	0.88	0.8	0.84	0.94	0.88	0.88	0.88	0.95	0.86	0.88	0.87	0.95
**miR-99b / miR-128a & miR-9* / miR-16 & miR-7 / miR-874**	0.84	0.88	0.86	0.95	0.92	0.88	0.9	0.97	0.9	0.86	0.88	0.95

Additional file 4. miRNA pairs and classifiers differentiating NDs from control in training, confirmation, and combined sets. A: AD vs. Control

SENS: Sensitivity, SPEC: Specificity, ACCUR: Accuracy, AUC: Area under ROC curve.

Set	Training				Confirmation				Combined			
	SENS	SPEC	ACCUR	AUC	SENS	SPEC	ACCUR	AUC	SENS	SPEC	ACCUR	AUC
**miR-9* / miR-181a**	0.8	0.52	0.66	0.69	0.84	0.8	0.82	0.88	0.64	0.68	0.66	0.76
**miR-9* / miR-874**	0.92	0.52	0.72	0.8	0.64	0.88	0.76	0.8	0.64	0.72	0.68	0.75
**miR-9* / miR-31**	0.68	0.64	0.66	0.68	0.6	0.76	0.68	0.75	0.56	0.7	0.63	0.7
**miR-9* / miR-125b**	0.6	0.76	0.68	0.76	0.76	0.84	0.8	0.81	0.72	0.58	0.65	0.75
**miR-9* / let-7e**	0.88	0.68	0.78	0.85	0.88	0.64	0.76	0.76	0.72	0.7	0.71	0.79
**miR-9* / miR-155**	0.76	0.72	0.74	0.8	0.72	0.92	0.82	0.85	0.7	0.72	0.71	0.82
**miR-9* / miR-9**	0.68	0.68	0.68	0.71	0.84	0.6	0.72	0.78	0.62	0.68	0.65	0.71
**miR-9* / miR-146a**	0.76	0.52	0.64	0.65	0.72	0.8	0.76	0.82	0.62	0.68	0.65	0.72
**miR-99b / miR-181a**	0.24	0.92	0.58	0.6	0.72	0.92	0.82	0.91	0.74	0.64	0.69	0.77
**miR-99b / let-7e**	0.68	0.76	0.72	0.83	0.56	0.92	0.74	0.76	0.74	0.74	0.74	0.77
**miR-7 / miR-874**	0.76	1	0.88	0.96	0.32	0.68	0.5	0.45	0.64	0.66	0.65	0.73
**miR-7 / miR-451**	0.84	0.72	0.78	0.89	0.72	0.6	0.66	0.64	0.64	0.66	0.65	0.75
**miR-323-3p / miR-134**	0.64	0.92	0.78	0.81	0.44	0.96	0.7	0.7	0.72	0.62	0.67	0.73
**miR-335-5p / miR-181a**	0.52	0.72	0.62	0.59	0.88	0.96	0.92	0.97	0.58	0.76	0.67	0.74
**miR-335-5p / let-7e**	0.52	0.92	0.72	0.8	0.76	1	0.88	0.94	0.76	0.62	0.69	0.83
**miR-335-5p / miR-155**	0.72	0.52	0.62	0.62	0.84	0.68	0.76	0.84	0.64	0.64	0.64	0.7
**miR-128a / miR-181a**	0.72	0.6	0.66	0.66	0.76	0.88	0.82	0.92	0.74	0.72	0.73	0.8
**miR-128a / miR-874**	0.84	0.48	0.66	0.71	0.8	0.6	0.7	0.72	0.56	0.68	0.62	0.7
**miR-128a / let-7e**	0.88	0.92	0.9	0.96	0.64	0.76	0.7	0.73	0.58	0.72	0.65	0.73
**miR-107 / let-7e**	0.8	0.84	0.82	0.87	0.64	0.48	0.56	0.52	0.76	0.58	0.67	0.71
**miR-338-3p / miR-181a**	0.72	0.8	0.76	0.78	0.72	0.6	0.66	0.72	0.62	0.64	0.63	0.71
**miR-191-5p / let-7e**	0.68	1	0.84	0.92	0.64	0.48	0.56	0.52	0.64	0.66	0.65	0.72
**miR-9* / let-7e & miR-335-5p / let-7e**	0.8	0.84	0.82	0.89	0.84	0.92	0.88	0.95	0.82	0.76	0.79	0.87
**miR-9* / miR-155 & miR-99b / let-7e**	0.92	0.8	0.86	0.89	0.6	0.96	0.78	0.86	0.72	0.86	0.79	0.87
**miR-9* / let-7e & miR-128a / miR-874**	0.8	1	0.9	0.96	0.68	0.88	0.78	0.87	0.74	0.94	0.84	0.91
**miR-9* / let-7e & miR-7 / miR-451 & miR-335-5p / let-7e**	0.84	0.96	0.9	0.97	0.84	0.96	0.9	0.97	0.86	0.88	0.87	0.94
**miR-9* / let-7e & miR-335-5p / let-7e & miR-128a / miR-874**	0.84	0.84	0.84	0.92	0.96	0.88	0.92	0.97	0.78	0.92	0.85	0.92
**miR-9* / miR-9 & miR-99b / let-7e & miR-335-5p / let-7e**	0.88	0.88	0.88	0.91	0.76	1	0.88	0.95	0.84	0.88	0.86	0.93

Aditional file 4. **B:** FTD vs. Control

Set	Training				Confirmation				Combined			
	SENS	SPEC	ACCUR	AUC	SENS	SPEC	ACCUR	AUC	SENS	SPEC	ACCUR	AUC
**miR-9* / miR-31**	0.67	0.64	0.65	0.74	0.88	0.92	0.9	0.98	0.82	0.74	0.78	0.85
**miR-9* / miR-138**	0.67	0.68	0.67	0.73	1	1	1	1	0.57	0.96	0.77	0.85
**miR-9* / miR-218**	0.63	0.6	0.61	0.65	0.88	0.96	0.92	0.98	0.59	0.92	0.76	0.83
**miR-9* / miR-129-3p**	0.63	0.58	0.6	0.76	0.96	1	0.98	1	0.8	0.82	0.81	0.91
**miR-9* / miR-874**	0.79	0.64	0.71	0.82	0.92	1	0.96	1	0.51	1	0.76	0.84
**miR-9* / miR-204**	0.58	0.56	0.57	0.67	1	1	1	1	0.76	0.9	0.83	0.87
**miR-9* / miR-29a**	0.54	0.44	0.49	0.5	1	1	1	1	0.53	0.98	0.76	0.82
**miR-99b / miR-31**	0.64	0.76	0.7	0.82	0.72	0.8	0.76	0.88	0.86	0.72	0.79	0.84
**miR-99b / miR-138**	0.68	0.76	0.72	0.79	1	0.96	0.98	0.99	0.84	0.86	0.85	0.9
**miR-99b / miR-218**	0.63	0.68	0.65	0.73	0.8	0.8	0.8	0.9	0.86	0.7	0.78	0.8
**miR-99b / miR-129-3p**	0.58	0.71	0.65	0.76	0.76	0.92	0.84	0.96	0.98	0.73	0.86	0.86
**miR-99b / miR-874**	0.76	0.72	0.74	0.86	0.92	0.84	0.88	0.97	0.8	0.8	0.8	0.9
**miR-99b / miR-125b**	0.6	0.6	0.6	0.74	0.92	0.84	0.88	0.95	0.86	0.72	0.79	0.85
**miR-99b / miR-204**	0.72	0.64	0.68	0.8	0.8	0.96	0.88	0.99	0.96	0.72	0.84	0.86
**miR-99b / miR-338-3p**	0.68	0.64	0.66	0.73	0.92	0.96	0.94	0.99	0.74	0.86	0.8	0.87
**miR-99b / miR-155**	0.64	0.68	0.66	0.74	0.96	0.8	0.88	0.99	0.92	0.7	0.81	0.88
**miR-99b / miR-29a**	0.68	0.68	0.68	0.73	0.92	0.76	0.84	0.96	0.78	0.76	0.77	0.81
**miR-99b / miR-181a**	0.52	0.44	0.48	0.53	0.88	0.96	0.92	0.99	0.58	0.94	0.76	0.8
**miR-99b / let-7e**	0.68	0.64	0.66	0.74	0.96	1	0.98	1	0.7	0.98	0.84	0.89
**miR-99b / miR-9**	0.64	0.56	0.6	0.68	0.92	0.96	0.94	0.99	0.78	0.78	0.78	0.86
**miR-99b / miR-146a**	0.6	0.52	0.56	0.66	1	0.88	0.94	0.99	0.76	0.7	0.73	0.82
**miR-99b / miR-128a**	0.6	0.6	0.6	0.66	0.88	0.92	0.9	0.98	0.56	0.94	0.75	0.8
**miR-127-3p / miR-411**	0.76	0.52	0.64	0.76	0.84	0.92	0.88	0.95	0.76	0.74	0.75	0.81
**miR-491-5p / miR-138**	0.76	0.72	0.74	0.85	0.76	0.6	0.68	0.81	0.72	0.68	0.7	0.81
**miR-128a / miR-138**	0.76	0.72	0.74	0.83	0.84	0.6	0.72	0.84	0.94	0.62	0.78	0.83
**miR-128a / miR-874**	0.8	0.84	0.82	0.92	0.64	0.64	0.64	0.75	0.76	0.72	0.74	0.81
**miR-335-5p / miR-138**	0.68	0.72	0.7	0.8	0.68	0.68	0.68	0.82	0.78	0.7	0.74	0.81
**miR-9* / miR-31 & miR-99b / miR-874**	0.92	0.56	0.74	0.82	0.96	0.92	0.94	0.99	0.94	0.7	0.82	0.91
**miR-99b / miR-874 & miR-127-3p / miR-411**	0.68	0.92	0.8	0.87	0.92	0.96	0.94	0.99	0.86	0.84	0.85	0.92
**miR-9* / miR-874 & miR-99b / miR-874 & miR-127-3p / miR-411**	0.68	1	0.84	0.9	0.92	1	0.96	1	0.84	0.94	0.89	0.95
**miR-9* / miR-129-3p & miR-99b / miR-874 & miR-127-3p / miR-411**	0.84	0.76	0.8	0.91	1	1	1	1	0.82	0.96	0.89	0.96
**miR-9* / miR-31 & miR-99b / miR-874 & miR-127-3p / miR-411**	0.88	0.72	0.8	0.87	0.96	0.96	0.96	0.99	0.9	0.84	0.87	0.94
**miR-9* / miR-129-3p & miR-127-3p / miR-411 & miR-491-5p / miR-138**	0.76	0.84	0.8	0.9	1	0.92	0.96	0.99	0.94	0.86	0.91	0.96

Additional file 4. **C:** PD vs. Control

Set	Training				Confirmation				Combined			
	SENS	SPEC	ACCUR	AUC	SENS	SPEC	ACCUR	AUC	SENS	SPEC	ACCUR	AUC
**miR-206 / miR-31**	0.8	0.8	0.8	0.91	0.8	0.84	0.82	0.92	0.86	0.78	0.82	0.9
**miR-206 / miR-129-3p**	0.84	0.79	0.82	0.92	0.75	0.76	0.76	0.88	0.82	0.82	0.82	0.88
**miR-206 / miR-138**	0.8	0.72	0.76	0.89	0.8	0.84	0.82	0.9	0.8	0.78	0.79	0.87
**miR-206 / miR-874**	0.76	0.84	0.8	0.89	0.76	0.84	0.8	0.9	0.78	0.8	0.79	0.86
**miR-206 / miR-125b**	0.8	0.76	0.78	0.89	0.8	0.84	0.82	0.9	0.74	0.84	0.79	0.87
**miR-206 / miR-204**	0.64	0.76	0.7	0.84	0.84	0.84	0.84	0.88	0.78	0.78	0.78	0.85
**miR-206 / miR-29a**	0.68	0.76	0.72	0.86	0.72	0.76	0.74	0.86	0.78	0.76	0.77	0.85
**miR-206 / miR-155**	0.72	0.72	0.72	0.83	0.72	0.72	0.72	0.84	0.8	0.72	0.76	0.82
**miR-206 / miR-338-3p**	0.64	0.72	0.68	0.81	0.72	0.72	0.72	0.85	0.7	0.74	0.72	0.82
**miR-206 / miR-16**	0.76	0.76	0.76	0.87	0.72	0.8	0.76	0.92	0.94	0.76	0.85	0.89
**miR-206 / miR-451**	0.76	0.76	0.76	0.85	0.84	0.72	0.78	0.92	0.72	0.84	0.78	0.87
**miR-206 / miR-218**	0.8	0.8	0.8	0.85	0.76	0.8	0.78	0.84	0.8	0.8	0.8	0.83
**miR-206 / miR-7**	0.64	0.76	0.7	0.81	0.68	0.76	0.72	0.88	0.92	0.56	0.74	0.82
**miR-99b / miR-129-3p**	0.68	0.58	0.63	0.64	0.79	0.88	0.84	0.94	0.76	0.73	0.74	0.79
**miR-99b / miR-204**	0.44	0.56	0.5	0.6	0.88	0.84	0.86	0.97	0.82	0.7	0.76	0.8
**miR-99b / miR-9**	0.56	0.52	0.54	0.58	0.88	0.96	0.92	0.95	0.78	0.7	0.74	0.81
**miR-99b / miR-155**	0.52	0.6	0.56	0.6	0.8	0.8	0.8	0.96	0.64	0.84	0.74	0.8
**miR-99b / miR-338-3p**	0.64	0.52	0.58	0.6	0.84	0.84	0.84	0.96	0.74	0.76	0.75	0.81
**miR-99b / let-7e**	0.67	0.44	0.55	0.52	0.92	0.84	0.88	0.98	0.73	0.72	0.73	0.79
**miR-9* / miR-129-3p**	0.48	0.46	0.47	0.63	0.88	0.96	0.92	0.96	0.51	1	0.76	0.8
**miR-335-5p / miR-338-3p**	0.72	0.72	0.72	0.81	0.72	0.8	0.76	0.84	0.62	0.74	0.68	0.78
**miR-206 / miR-129-3p & miR-335-5p / miR-338-3p**	0.68	1	0.84	0.93	0.84	0.88	0.86	0.95	0.78	0.78	0.78	0.92
**miR-206 / miR-31 & miR-335-5p / miR-338-3p**	0.64	1	0.82	0.93	0.84	0.92	0.88	0.96	0.84	0.92	0.88	0.96
**miR-206 / miR-874 & miR-335-5p / miR-338-3p**	0.64	1	0.82	0.91	0.76	0.96	0.86	0.95	0.78	0.96	0.87	0.94
**miR-206 / miR-16 & miR-335-5p / miR-338-3p**	0.76	0.88	0.82	0.9	0.76	0.96	0.86	0.95	0.72	0.88	0.8	0.91
**miR-206 / miR-204 & miR-206 / miR-218 & miR-335-5p / miR-338-3p**	0.64	0.92	0.78	0.88	0.92	0.8	0.86	0.87	0.88	0.74	0.81	0.91
**miR-206 / miR-204 & miR-206 / miR-218 & miR-9* / miR-129-3p**	0.8	0.8	0.8	0.85	0.84	0.96	0.9	0.98	0.88	0.82	0.85	0.91
**miR-206 / miR-338-3p & miR-99b / miR-129-3p & miR-335-5p / miR-338-3p**	0.68	0.84	0.76	0.88	0.96	0.92	0.94	0.98	0.84	0.8	0.82	0.92
**miR-206 / miR-338-3p & miR-99b / miR-155 & miR-335-5p / miR-338-3p**	0.68	0.92	0.8	0.88	0.88	0.96	0.92	0.98	0.76	0.9	0.83	0.93

Additional file 4. **D:** ALS vs. Control
