# Supplementary material for: Circulating brain-enriched microRNAs as novel biomarkers for detection and differentiation of neurodegenerative diseases
Source: Alzheimers Res Ther. 2017 Nov 9;9:89. doi: 10.1186/s13195-017-0316-0 (PMC5679501; doi:10.1186/s13195-017-0316-0)
Supplement: Supplementary file 6 — miRNA pairs and classifiers differentiating NDs from each other in training, confirmation, and combined sets. (PDF 291 kb) [file 13195_2017_316_MOESM6_ESM.pdf]

Set	Training				Confirmation				Combined			
	SENS	SPEC	ACCUR	AUC	SENS	SPEC	ACCUR	AUC	SENS	SPEC	ACCUR	AUC
**miR-125b / miR-29a**	0.64	0.68	0.66	0.83	0.72	0.72	0.72	0.8	0.72	0.78	0.75	0.78
**miR-125b / miR-874**	0.68	0.68	0.68	0.82	0.72	0.6	0.66	0.7	0.7	0.66	0.68	0.75
**miR-107 / miR-335-5p**	0.72	0.68	0.7	0.72	0.8	0.8	0.8	0.91	0.7	0.74	0.72	0.8
**miR-155 / miR-29a**	0.76	0.64	0.7	0.77	0.76	0.64	0.7	0.78	0.66	0.66	0.66	0.75
**miR-125b / miR-29a & miR-107 / miR-335-5p& miR-155 / miR-29a**	0.76	0.84	0.8	0.87	0.88	0.88	0.88	0.94	0.78	0.74	0.76	0.87
**miR-125b / miR-29a & miR-125b / miR-874 & miR-107 / miR-335-5p**	0.64	0.68	0.66	0.85	0.84	0.84	0.84	0.92	0.78	0.76	0.77	0.87
**miR-125b / miR-874 & miR-107 / miR-335-5p& miR-155 / miR-29a**	0.76	0.84	0.8	0.87	0.88	0.88	0.88	0.94	0.76	0.76	0.76	0.87

Additional file 6. **miRNA pairs and classifiers differentiating NDs from each other in training, confirmation, and combined sets. A:** AD vs. FTD

SENS: Sensitivity, SPEC: Specificity, ACCUR: Accuracy, AUC: Area under ROC curve.

Set	Training				Confirmation				Combined			
	SENS	SPEC	ACCUR	AUC	SENS	SPEC	ACCUR	AUC	SENS	SPEC	ACCUR	AUC
**miR-206 / miR-9***	0.56	0.54	0.55	0.6	0.84	0.84	0.84	0.94	0.72	0.71	0.72	0.77
**miR-31 / miR-9***	0.56	0.63	0.59	0.62	0.96	0.88	0.92	0.98	0.72	0.71	0.72	0.8
**miR-31 / miR-99b**	0.76	0.64	0.7	0.76	0.72	0.64	0.68	0.82	0.76	0.68	0.72	0.77
**miR-31 / miR-16**	0.72	0.8	0.76	0.82	0.72	0.72	0.72	0.76	0.68	0.76	0.72	0.77
**miR-31 / miR-29a**	0.68	0.6	0.64	0.79	0.8	0.72	0.76	0.85	0.8	0.66	0.73	0.81
**miR-138 / miR-9***	0.44	0.5	0.47	0.54	1	1	1	1	0.7	0.65	0.68	0.76
**miR-138 / miR-99b**	0.64	0.56	0.6	0.7	0.84	0.8	0.82	0.93	0.74	0.68	0.71	0.82
**miR-138 / miR-181a**	0.76	0.72	0.74	0.84	0.72	0.68	0.7	0.81	0.64	0.72	0.68	0.78
**miR-138 / miR-491-5p**	0.72	0.68	0.7	0.78	0.64	0.72	0.68	0.78	0.7	0.7	0.7	0.77
**miR-138 / miR-335-5p**	0.68	0.68	0.68	0.76	0.64	0.68	0.66	0.83	0.64	0.74	0.69	0.79
**miR-138 / miR-128a**	0.68	0.72	0.7	0.81	0.64	0.56	0.6	0.8	0.7	0.7	0.7	0.78
**miR-138 / miR-146a**	0.6	0.68	0.64	0.71	0.8	0.72	0.76	0.83	0.6	0.74	0.67	0.77
**miR-138 / let-7e**	0.72	0.72	0.72	0.83	0.68	0.6	0.64	0.74	0.66	0.7	0.68	0.77
**miR-138 / miR-16**	0.64	0.68	0.66	0.72	0.8	0.68	0.74	0.87	0.72	0.62	0.67	0.77
**miR-138 / miR-29a**	0.52	0.48	0.5	0.61	0.8	0.8	0.8	0.91	0.72	0.66	0.69	0.76
**miR-129-3p / miR-9***	0.68	0.58	0.63	0.75	1	0.92	0.96	0.99	0.7	0.73	0.72	0.85
**miR-129-3p / miR-99b**	0.8	0.58	0.69	0.83	0.8	0.68	0.74	0.86	0.76	0.76	0.76	0.83
**miR-129-3p / miR-335-5p**	0.72	0.83	0.78	0.88	0.6	0.6	0.6	0.7	0.68	0.63	0.66	0.78
**miR-129-3p / miR-146a**	0.76	0.67	0.71	0.85	0.6	0.68	0.64	0.7	0.72	0.55	0.64	0.77
**miR-129-3p / miR-16**	0.8	0.79	0.8	0.85	0.68	0.56	0.62	0.72	0.74	0.67	0.71	0.78
**miR-129-3p / miR-29a**	0.64	0.67	0.65	0.8	0.64	0.6	0.62	0.77	0.62	0.65	0.64	0.76
**miR-125b / miR-9***	0.48	0.63	0.55	0.62	0.96	0.96	0.96	0.98	0.74	0.61	0.68	0.75
**miR-125b / miR-99b**	0.64	0.6	0.62	0.75	0.76	0.76	0.76	0.88	0.78	0.66	0.72	0.81
**miR-125b / miR-181a**	0.76	0.84	0.8	0.93	0.68	0.64	0.66	0.73	0.66	0.76	0.71	0.78
**miR-125b / miR-491-5p**	0.84	0.84	0.84	0.9	0.56	0.76	0.66	0.71	0.7	0.8	0.75	0.79
**miR-125b / miR-335-5p**	0.76	0.76	0.76	0.86	0.68	0.72	0.7	0.77	0.68	0.72	0.7	0.8
**miR-125b / miR-128a**	0.8	0.8	0.8	0.92	0.6	0.8	0.7	0.74	0.7	0.78	0.74	0.8
**miR-125b / miR-146a**	0.6	0.76	0.68	0.84	0.56	0.76	0.66	0.8	0.7	0.68	0.69	0.78
**miR-125b / let-7e**	0.84	0.88	0.86	0.95	0.52	0.72	0.62	0.65	0.74	0.78	0.76	0.79
**miR-218 / miR-9***	0.56	0.63	0.59	0.61	0.84	0.88	0.86	0.96	0.62	0.73	0.68	0.77
**miR-218 / miR-16**	0.64	0.71	0.67	0.77	0.68	0.72	0.7	0.79	0.7	0.71	0.71	0.76
**miR-204 / miR-9***	0.56	0.58	0.57	0.66	0.84	1	0.92	1	0.72	0.76	0.74	0.82
**miR-204 / miR-99b**	0.72	0.72	0.72	0.79	0.84	0.68	0.76	0.89	0.68	0.66	0.67	0.8
**miR-204 / miR-146a**	0.76	0.68	0.72	0.86	0.6	0.72	0.66	0.76	0.76	0.64	0.7	0.78
**miR-338-3p / miR-99b**	0.52	0.6	0.56	0.64	0.76	0.8	0.78	0.88	0.66	0.66	0.66	0.75
**miR-107 / miR-99b**	0.36	0.64	0.5	0.53	0.92	0.92	0.92	0.99	0.66	0.72	0.69	0.78
**miR-107 / miR-491-5p**	0.72	0.56	0.64	0.76	0.72	0.6	0.66	0.79	0.72	0.56	0.64	0.76
**miR-31 / miR-16 & miR-129-3p / miR-9* & miR-204 / miR-99b**	0.8	0.92	0.86	0.91	0.96	0.8	0.88	0.98	0.88	0.88	0.88	0.95
**miR-129-3p / miR-16 & miR-125b / miR-181a & miR-204 / miR-9***	0.8	0.84	0.82	0.95	0.8	0.88	0.84	0.96	0.84	0.86	0.85	0.95
**miR-31 / miR-99b & miR-129-3p / miR-16 & miR-204 / miR-9***	0.8	0.76	0.78	0.93	0.92	0.8	0.86	0.98	0.9	0.88	0.89	0.96
**miR-31 / miR-16 & miR-125b / miR-181a & miR-204 / miR-9***	0.8	0.84	0.82	0.94	0.84	0.84	0.84	0.96	0.82	0.88	0.85	0.95
**miR-31 / miR-16 & miR-129-3p / miR-335-5p & miR-204 / miR-9***	0.88	0.88	0.88	0.94	0.84	0.84	0.84	0.96	0.86	0.9	0.88	0.96

Additional file 6. **B:** AD vs. PD

Set	Training				Confirmation				Combined			
	SENS	SPEC	ACCUR	AUC	SENS	SPEC	ACCUR	AUC	SENS	SPEC	ACCUR	AUC
**miR-329 / miR-206**	0.8	0.8	0.8	0.89	0.72	0.68	0.7	0.77	0.76	0.76	0.76	0.82
**miR-329 / miR-9***	0.68	0.64	0.66	0.73	0.8	0.72	0.76	0.83	0.68	0.58	0.63	0.77
**miR-329 / miR-99b**	0.68	0.72	0.7	0.75	0.76	0.68	0.72	0.86	0.72	0.7	0.71	0.78
**miR-329 / miR-335-5p**	0.84	0.72	0.78	0.89	0.68	0.6	0.64	0.7	0.74	0.66	0.7	0.79
**miR-329 / miR-146a**	0.8	0.76	0.78	0.87	0.6	0.56	0.58	0.67	0.74	0.64	0.69	0.77
**miR-329 / miR-411**	0.76	0.72	0.74	0.84	0.68	0.56	0.62	0.67	0.74	0.64	0.69	0.76
**miR-31 / miR-206**	0.84	0.76	0.8	0.87	0.88	0.8	0.84	0.91	0.78	0.78	0.78	0.88
**miR-31 / miR-16**	0.64	0.6	0.62	0.71	0.8	0.68	0.74	0.87	0.7	0.7	0.7	0.78
**miR-129-3p / miR-206**	0.88	0.84	0.86	0.92	0.72	0.79	0.76	0.85	0.8	0.82	0.81	0.87
**miR-129-3p / miR-16**	0.72	0.64	0.68	0.8	0.68	0.67	0.67	0.75	0.74	0.61	0.68	0.76
**miR-433 / miR-206**	0.8	0.76	0.78	0.85	0.56	0.76	0.66	0.8	0.7	0.74	0.72	0.8
**miR-433 / miR-335-5p**	0.76	0.56	0.66	0.79	0.72	0.72	0.72	0.76	0.76	0.6	0.68	0.76
**miR-125b / miR-206**	0.8	0.8	0.8	0.91	0.8	0.8	0.8	0.88	0.76	0.78	0.77	0.87
**miR-125b / miR-335-5p**	0.64	0.68	0.66	0.8	0.68	0.76	0.72	0.78	0.62	0.72	0.67	0.78
**miR-125b / miR-874**	0.72	0.8	0.76	0.9	0.68	0.52	0.6	0.64	0.7	0.7	0.7	0.75
**miR-323-3p / miR-206**	0.8	0.8	0.8	0.85	0.68	0.72	0.7	0.78	0.7	0.72	0.71	0.8
**miR-495 / miR-206**	0.76	0.76	0.76	0.84	0.68	0.72	0.7	0.78	0.72	0.76	0.74	0.79
**miR-382 / miR-206**	0.72	0.68	0.7	0.82	0.64	0.68	0.66	0.77	0.68	0.72	0.7	0.77
**miR-204 / miR-206**	0.6	0.76	0.68	0.85	0.72	0.76	0.74	0.82	0.76	0.74	0.75	0.82
**miR-127-3p / miR-206**	0.8	0.72	0.76	0.81	0.64	0.72	0.68	0.78	0.7	0.72	0.71	0.78
**miR-107 / miR-206**	0.76	0.76	0.76	0.86	0.68	0.68	0.68	0.75	0.74	0.68	0.71	0.79
**miR-107 / miR-99b**	0.4	0.72	0.56	0.59	0.8	0.84	0.82	0.88	0.68	0.74	0.71	0.77
**miR-107 / miR-335-5p**	0.72	0.8	0.76	0.9	0.68	0.64	0.66	0.77	0.74	0.62	0.68	0.82
**miR-107 / let-7e**	0.96	0.83	0.9	0.98	0.36	0.52	0.44	0.48	0.7	0.61	0.66	0.79
**miR-107 / miR-146a**	0.72	0.72	0.72	0.85	0.68	0.68	0.68	0.81	0.76	0.78	0.77	0.83
**miR-107 / miR-491-5p**	0.88	0.8	0.84	0.88	0.68	0.68	0.68	0.75	0.74	0.7	0.72	0.8
**miR-138 / miR-206**	0.72	0.76	0.74	0.84	0.8	0.8	0.8	0.89	0.76	0.7	0.73	0.84
**miR-411 / miR-206**	0.76	0.72	0.74	0.83	0.6	0.72	0.66	0.75	0.66	0.74	0.7	0.77
**miR-134 / miR-206**	0.76	0.68	0.72	0.8	0.64	0.64	0.64	0.76	0.66	0.72	0.69	0.76
**miR-9 / miR-206**	0.72	0.76	0.74	0.82	0.64	0.68	0.66	0.75	0.72	0.7	0.71	0.76
**miR-155 / miR-206**	0.68	0.76	0.72	0.83	0.76	0.72	0.74	0.8	0.74	0.72	0.73	0.8
**miR-7 / miR-206**	0.72	0.64	0.68	0.85	0.64	0.68	0.66	0.84	0.7	0.7	0.7	0.81
**miR-338-3p / miR-206**	0.76	0.8	0.78	0.86	0.64	0.68	0.66	0.77	0.76	0.66	0.71	0.8
**miR-29a / miR-206**	0.88	0.8	0.84	0.9	0.68	0.72	0.7	0.77	0.76	0.78	0.77	0.82
**miR-31 / miR-206 &<br>miR-125b / miR-335-5p**	0.96	0.8	0.88	0.95	0.84	0.88	0.86	0.96	0.86	0.8	0.83	0.93
**miR-31 / miR-206 &<br>miR-125b / miR-335-5p &<br>miR-107 / miR-335-5p**	1	0.92	0.96	0.99	0.88	0.96	0.92	0.97	0.94	0.92	0.93	0.98
**miR-31 / miR-206 &<br>miR-125b / miR-335-5p &<br>miR-107 / miR-491-5p**	0.92	0.88	0.9	0.99	0.92	0.96	0.94	0.98	0.92	0.94	0.93	0.98
**miR-125b / miR-335-5p &<br>miR-107 / let-7e &<br>miR-138 / miR-206**	1	0.96	0.98	1	0.84	0.8	0.82	0.94	0.9	0.9	0.9	0.98
**miR-125b / miR-335-5p &<br>miR-107 / miR-491-5p &<br>miR-338-3p / miR-206**	0.88	1	0.94	1	0.84	0.92	0.88	0.96	0.92	0.94	0.93	0.98
**miR-125b / miR-335-5p &<br>miR-107 / let-7e &<br>miR-338-3p / miR-206**	1	1	1	1	0.8	0.88	0.84	0.87	0.94	0.9	0.92	0.98
**miR-125b / miR-335-5p &<br>miR-323-3p / miR-206 &<br>miR-107 / let-7e**	1	0.96	0.98	1	0.8	0.88	0.84	0.89	0.94	0.88	0.91	0.98

Additional file 6. **C:** AD vs. ALS

Set	Training				Confirmation				Combined			
	SENS	SPEC	ACCUR	AUC	SENS	SPEC	ACCUR	AUC	SENS	SPEC	ACCUR	AUC
**miR-218 / miR-9***	0.64	0.63	0.63	0.71	0.84	0.76	0.8	0.92	0.66	0.69	0.68	0.78
**miR-218 / miR-99b**	0.76	0.67	0.71	0.8	0.6	0.68	0.64	0.74	0.68	0.67	0.68	0.76
**miR-138 / miR-99b**	0.68	0.64	0.66	0.77	0.68	0.68	0.68	0.87	0.72	0.68	0.7	0.79
**miR-138 / miR-181a**	0.68	0.68	0.68	0.82	0.56	0.68	0.62	0.73	0.62	0.72	0.67	0.77
**miR-138 / miR-491-5p**	0.72	0.72	0.72	0.84	0.6	0.68	0.64	0.69	0.68	0.7	0.69	0.75
**miR-31 / miR-9***	0.64	0.54	0.59	0.67	0.8	0.8	0.8	0.94	0.7	0.59	0.65	0.76
**miR-31 / miR-99b**	0.72	0.64	0.68	0.79	0.68	0.56	0.62	0.78	0.68	0.6	0.64	0.77
**miR-129-3p / miR-9***	0.52	0.54	0.53	0.59	0.88	0.92	0.9	0.95	0.68	0.67	0.68	0.8
**miR-129-3p / miR-99b**	0.72	0.58	0.65	0.8	0.8	0.64	0.72	0.84	0.72	0.73	0.73	0.8
**miR-204 / miR-9***	0.52	0.54	0.53	0.62	0.8	0.96	0.88	0.94	0.76	0.71	0.74	0.77
**miR-204 / miR-99b**	0.68	0.72	0.7	0.83	0.68	0.6	0.64	0.83	0.68	0.66	0.67	0.81
**miR-338-3p / miR-99b**	0.68	0.68	0.68	0.8	0.84	0.88	0.86	0.94	0.74	0.78	0.76	0.86
**miR-338-3p / miR-491-5p**	0.84	0.8	0.82	0.92	0.6	0.68	0.64	0.77	0.7	0.76	0.73	0.84
**miR-338-3p / miR-107**	0.72	0.8	0.76	0.88	0.6	0.72	0.66	0.7	0.68	0.74	0.71	0.78
**miR-338-3p / let-7e**	0.76	0.8	0.78	0.88	0.52	0.56	0.54	0.62	0.58	0.68	0.63	0.76
**miR-338-3p / miR-146a**	0.68	0.76	0.72	0.84	0.76	0.68	0.72	0.79	0.66	0.72	0.69	0.79
**miR-874 / miR-99b**	0.68	0.72	0.7	0.77	0.72	0.6	0.66	0.8	0.7	0.64	0.67	0.77
**miR-125b / miR-99b**	0.6	0.6	0.6	0.73	0.72	0.76	0.74	0.83	0.68	0.62	0.65	0.77
**miR-29a / miR-99b**	0.76	0.72	0.74	0.81	0.76	0.72	0.74	0.83	0.72	0.76	0.74	0.79
**miR-323-3p / miR-127-3p**	0.76	0.76	0.76	0.87	0.68	0.56	0.62	0.68	0.68	0.74	0.71	0.78
**miR-411 / miR-127-3p**	0.76	0.76	0.76	0.85	0.64	0.64	0.64	0.76	0.6	0.76	0.68	0.8
**miR-155 / miR-99b**	0.72	0.56	0.64	0.66	0.8	0.76	0.78	0.88	0.76	0.66	0.71	0.76
**miR-335-5p / miR-99b**	0.4	0.56	0.48	0.53	0.8	1	0.9	0.96	0.64	0.7	0.67	0.76
**miR-129-3p / miR-9* & miR-338-3p / miR-99b & miR-323-3p / miR-127-3p**	0.6	0.8	0.7	0.88	0.84	1	0.92	0.97	0.84	0.9	0.87	0.93
**miR-129-3p / miR-9* & miR-338-3p / miR-491-5p& miR-411 / miR-127-3p**	0.76	0.8	0.78	0.9	0.84	0.88	0.86	0.94	0.78	0.9	0.84	0.93
**miR-129-3p / miR-9* & miR-338-3p / miR-491-5p& miR-323-3p / miR-127-3p**	0.72	0.76	0.74	0.91	0.76	0.84	0.8	0.91	0.76	0.88	0.82	0.93
**miR-204 / miR-9* & miR-338-3p / miR-491-5p& miR-323-3p / miR-127-3p**	0.8	0.8	0.8	0.9	0.8	0.88	0.84	0.93	0.74	0.88	0.81	0.92
**miR-129-3p / miR-9* & miR-29a / miR-99b & miR-323-3p / miR-127-3p**	0.68	0.8	0.74	0.88	0.8	0.92	0.86	0.93	0.74	0.84	0.79	0.93
**miR-138 / miR-181a & miR-129-3p / miR-9* & miR-411 / miR-127-3p**	0.6	0.84	0.72	0.86	0.8	0.92	0.86	0.93	0.74	0.94	0.84	0.92

Additional file 6. **D:** FTD vs. PD

Set	Training				Confirmation				Combined			
	SENS	SPEC	ACCUR	AUC	SENS	SPEC	ACCUR	AUC	SENS	SPEC	ACCUR	AUC
**miR-31 / miR-206**	0.8	0.76	0.78	0.87	0.76	0.84	0.8	0.92	0.8	0.76	0.78	0.87
**miR-138 / miR-206**	0.76	0.92	0.84	0.89	0.84	0.76	0.8	0.87	0.78	0.7	0.74	0.85
**miR-29a / miR-206**	0.84	0.8	0.82	0.9	0.76	0.72	0.74	0.87	0.78	0.78	0.78	0.87
**miR-129-3p / miR-206**	0.76	0.68	0.72	0.85	0.8	0.83	0.82	0.91	0.8	0.8	0.8	0.86
**miR-204 / miR-206**	0.76	0.76	0.76	0.84	0.76	0.84	0.8	0.89	0.74	0.74	0.74	0.85
**miR-338-3p / miR-206**	0.72	0.76	0.74	0.87	0.72	0.72	0.72	0.83	0.74	0.72	0.73	0.84
**miR-338-3p / let-7e**	0.96	0.83	0.9	0.99	0.44	0.56	0.5	0.55	0.58	0.76	0.67	0.81
**miR-7 / miR-206**	0.88	0.88	0.88	0.95	0.64	0.68	0.66	0.84	0.76	0.68	0.72	0.85
**miR-128a / miR-206**	0.72	0.76	0.74	0.85	0.72	0.72	0.72	0.83	0.76	0.72	0.74	0.82
**miR-16 / miR-206**	0.8	0.8	0.8	0.94	0.76	0.68	0.72	0.86	0.76	0.76	0.76	0.88
**miR-125b / miR-206**	0.72	0.8	0.76	0.86	0.76	0.76	0.76	0.89	0.78	0.74	0.76	0.85
**miR-874 / miR-206**	0.8	0.72	0.76	0.85	0.8	0.76	0.78	0.88	0.72	0.78	0.75	0.84
**miR-218 / miR-206**	0.8	0.84	0.82	0.88	0.8	0.68	0.74	0.82	0.76	0.8	0.78	0.83
**miR-29a / miR-206 & miR-338-3p / let-7e**	0.96	0.92	0.94	1	0.64	0.76	0.7	0.78	0.8	0.84	0.82	0.93
**miR-129-3p / miR-206 & miR-338-3p / let-7e**	0.92	0.88	0.9	0.99	0.68	0.72	0.7	0.8	0.84	0.86	0.85	0.94
**miR-204 / miR-206 & miR-338-3p / let-7e**	0.92	0.96	0.94	0.99	0.6	0.72	0.66	0.81	0.8	0.88	0.84	0.94
**miR-138 / miR-206 & miR-338-3p / let-7e**	0.92	0.96	0.94	1	0.6	0.8	0.7	0.78	0.78	0.9	0.84	0.93
**miR-129-3p / miR-206 & miR-338-3p / let-7e & miR-125b / miR-206**	0.88	0.84	0.86	0.96	0.6	0.8	0.7	0.89	0.84	0.82	0.83	0.93
**miR-129-3p / miR-206 & miR-338-3p / let-7e & miR-874 / miR-206**	0.88	0.8	0.84	0.96	0.64	0.8	0.72	0.87	0.84	0.82	0.83	0.93
**miR-129-3p / miR-206 & miR-338-3p / let-7e & miR-7 / miR-206**	0.92	0.84	0.88	0.99	0.68	0.8	0.74	0.84	0.8	0.82	0.81	0.93

Additional file 6. **E:** FTD vs. ALS

Set	Training				Confirmation				Combined			
	SENS	SPEC	ACCUR	AUC	SENS	SPEC	ACCUR	AUC	SENS	SPEC	ACCUR	AUC
**miR-329 / miR-206**	0.68	0.72	0.7	0.83	0.72	0.76	0.74	0.87	0.78	0.76	0.77	0.84
**miR-9* / miR-206**	0.72	0.67	0.69	0.89	0.84	0.84	0.84	0.92	0.78	0.78	0.78	0.87
**miR-433 / miR-206**	0.72	0.8	0.76	0.84	0.76	0.68	0.72	0.88	0.74	0.76	0.75	0.85
**miR-370 / miR-206**	0.72	0.72	0.72	0.84	0.76	0.72	0.74	0.84	0.68	0.72	0.7	0.81
**miR-134 / miR-206**	0.68	0.76	0.72	0.84	0.76	0.72	0.74	0.87	0.72	0.74	0.73	0.84
**miR-323-3p / miR-206**	0.68	0.76	0.72	0.84	0.76	0.68	0.72	0.87	0.76	0.76	0.76	0.85
**miR-9 / miR-206**	0.76	0.72	0.74	0.87	0.76	0.72	0.74	0.86	0.7	0.78	0.74	0.85
**miR-99b / miR-206**	0.8	0.76	0.78	0.9	0.72	0.76	0.74	0.84	0.74	0.8	0.77	0.86
**miR-495 / miR-206**	0.72	0.72	0.72	0.82	0.76	0.72	0.74	0.87	0.76	0.76	0.76	0.84
**miR-382 / miR-206**	0.68	0.76	0.72	0.85	0.76	0.68	0.72	0.85	0.72	0.72	0.72	0.84
**miR-491-5p / miR-206**	0.92	0.64	0.78	0.92	0.76	0.72	0.74	0.87	0.76	0.76	0.76	0.87
**miR-29a / miR-206**	0.72	0.8	0.76	0.9	0.8	0.72	0.76	0.9	0.76	0.78	0.77	0.89
**miR-411 / miR-206**	0.72	0.72	0.72	0.84	0.76	0.68	0.72	0.86	0.7	0.74	0.72	0.84
**miR-181a / miR-206**	0.76	0.68	0.72	0.88	0.72	0.68	0.7	0.86	0.76	0.72	0.74	0.84
**miR-155 / miR-206**	0.8	0.76	0.78	0.89	0.72	0.76	0.74	0.87	0.8	0.74	0.77	0.87
**miR-146a / miR-206**	0.64	0.76	0.7	0.88	0.76	0.76	0.76	0.86	0.76	0.68	0.72	0.86
**miR-7 / miR-206**	0.72	0.88	0.8	0.92	0.88	0.72	0.8	0.89	0.8	0.76	0.78	0.89
**miR-29a / miR-206 & miR-7 / miR-206**	0.88	0.76	0.82	0.92	0.72	0.76	0.74	0.9	0.78	0.76	0.77	0.9
**miR-9* / miR-206 & miR-7 / miR-206**	0.8	0.76	0.78	0.91	0.8	0.84	0.82	0.92	0.78	0.8	0.79	0.91
**miR-9* / miR-206 & miR-155 / miR-206 & miR-7 / miR-206**	0.8	0.8	0.8	0.9	0.84	0.8	0.82	0.91	0.82	0.8	0.81	0.9

Additional file 6. **F:** PD vs. ALS
