# Supplementary material for: Circulating brain-enriched microRNAs as novel biomarkers for detection and differentiation of neurodegenerative diseases
Source: Alzheimers Res Ther. 2017 Nov 9;9:89. doi: 10.1186/s13195-017-0316-0 (PMC5679501; doi:10.1186/s13195-017-0316-0)
Supplement: Supplementary file 8 — Differentiation of ND subgroups from other research participants with the same ND. (PDF 89 kb) [file 13195_2017_316_MOESM8_ESM.pdf]

A. Differentiation of FTD syndromes from other FTD patients. bv – behavioral variant (23 participants); PPA – primary progressive aphasia; (17 participants) PSP – progressive supranuclear palsy (10 participants).

Subgroup (N)		SENS	SPEC	ACCUR	AUC
FTD_bv (23)	**miR-335-5p / miR-433**	0.59	0.74	0.66	0.71
	**miR-335-5p / miR-370**	0.48	0.74	0.6	0.72
	**miR-29a / miR-204**	0.7	0.61	0.66	0.71
	**let-7e / miR-370**	0.33	0.74	0.52	0.74
	**miR-335-5p / miR-433 + miR-335-5p / miR-370 + miR-29a / miR-204**	0.44	0.78	0.6	0.77
FTD_PPA (17)	**miR-370 / miR-29a**	0.7	0.47	0.62	0.7
	**miR-134 / miR-29a**	0.67	0.53	0.62	0.72
	**miR-181a / miR-138**	0.61	0.59	0.6	0.71
	**miR-338-3p / miR-29a**	0.58	0.65	0.6	0.7
	**miR-134 / miR-29a + miR-181a / miR-138 + miR-338-3p / miR-29a**	0.73	0.71	0.72	0.76
FTD_PSP (10)	**miR-204 / miR-9**	0.65	0.4	0.6	0.74
	**miR-323-3p / miR-134**	0.7	0.5	0.66	0.74
	**miR-155 / miR-99b**	0.75	0.7	0.74	0.74
	**miR-338-3p / miR-181a**	0.75	0.7	0.74	0.77
	**miR-338-3p / miR-9 + miR-323-3p /miR-411 + miR-323-3p / miR-370**	0.8	0.5	0.74	0.87

Additional file 8. Differentiation of ND subgroups from other research participants with the same ND

The data is given for the combined groups (total 50 participants for each ND). For example, the data for FTD_PPA presents miRNA pairs and classifiers differentiating FTD patients with primary progressive aphasia (17 participants) from all other FTD patients (33 participants). SENS: Sensitivity, SPEC: Specificity, ACCUR: Accuracy, AUC: Area under ROC curve.

B. Differentiation of PD with dementia (12 participants) from other PD patients.

Subgroup (N)		SENS	SPEC	ACCUR	AUC
PD_DEM (12)	**miR-7 / miR-146a**	0.63	0.75	0.66	0.77
	**miR-206 / miR-9**	0.74	0.58	0.7	0.76
	**miR-7 / miR-9**	0.66	0.75	0.68	0.81
	**miR-7 / miR-128a**	0.71	0.67	0.7	0.79
	**miR-107 / miR-9**	0.61	0.75	0.64	0.79
	**miR-7 / miR-146a + miR-7 / miR-9 + miR-107 / miR- 9**	0.71	0.67	0.7	0.84

C. Differentiation of ALS-FTD (8 participants) from other ALS patients.

Subgroup (N)		SENS	SPEC	ACCUR	AUC
ALS-FTD (8)	**miR-129-3p / miR-874**	0.66	0.75	0.67	0.76
	**miR-29a / miR-9***	0.57	0.63	0.58	0.78
	**miR-146a / miR-9**	0.62	0.75	0.64	0.78
	**miR-491-5p / let-7e**	0.61	0.63	0.61	0.76
	**miR-218 / miR-451**	0.57	0.63	0.58	0.75
	**miR-218 / miR-451 + miR-29a / miR-9* + miR-491-5p / let-7e**	0.74	0.88	0.76	0.96

Additional file 8. Differentiation of ND subgroups from other research participants with the same ND
